# Nutritional supplement products: does the label information influence purchasing decisions for the physically active?

**DOI:** 10.1186/1475-2891-12-133

**Published:** 2013-10-02

**Authors:** Gary Gabriels, Mike Lambert

**Affiliations:** 1 Department of Medicine, Division of Clinical Pharmacology, Faculty of Health Sciences, Observatory, University of Cape Town, Cape Town 7925, South Africa; 2 Department of Human BiologyMedical Research Council/University of Cape Town Research Unit for Exercise Science and Sports Medicine, University of Cape Town, Cape Town, South Africa

**Keywords:** Nutritional supplements, Information labels, Consumers, Olympics, Paralympics, Physical activity, Laboratory screen testing

## Abstract

**Background:**

The increase in sales of nutritional supplement globally can be attributed, in part, to aggressive marketing by manufacturers, rather than because the nutritional supplements have become more effective. Furthermore, the accuracy of the labelling often goes unchallenged. Therefore, any effects of the supplement, may be due to contaminants or adulterants in these products not reflected on the label.

**Methods:**

A self-administered questionnaire was used to determine how consumers of nutritional supplements acquired information to assist their decision-making processes, when purchasing a product. The study was approved by the University of Cape Town, Faculty of Health Sciences Human Research Ethics Committee. The questionnaire consisted of seven, closed and open-ended questions. The participants were asked to respond to the questions according to a defined list of statements. A total of 259 participants completed and returned questionnaires. The data and processing of the returned questionnaires was captured using Windows-based Microsoft® Office Excel 2003 SP 1 (Excel © 1985–2003 Microsoft Corporation). Statistica Version 10 (copyright © Stat Soft, Inc. 1984–2011) was used to calculate the descriptive statistics.

**Results:**

The main finding of the study was that nearly 70% of the respondents who purchased supplements were strongly influenced by container label information that stipulated that the nutritional supplement product is free of banned substances. The second finding was that just over 50% of the respondents attached importance to the quality of the nutritional supplement product information on the container label. The third finding was that about 40% of the respondents were strongly influenced by the ingredients on the labels when they purchased nutritional supplements.

**Conclusion:**

This study, (i) identifies short-comings in current labelling information practices, (ii) provides opportunities to improve label and non-label information and communication, and, (iii) presents the case for quality assurance laboratory “screening testing” of declared and undeclared contaminants and/or adulterants, that could have negative consequences to the consumer.

## Background

The nutritional supplements (nutraceuticals) sector is generally encumbered with statutory laws in two extremes. Notably, those laws that govern medicines and those laws that govern foods. Legislation (statutory laws) is law promulgated by a legislature or a governing body. Regulations on the other hand are measures to control human or societal behaviour by rules or restrictions. Regulations, can take the form of legal restrictions or self-regulation. As such, medicine and food production, processing, distribution, retail, packaging and labelling in general is a multifaceted industry often governed by several laws, regulations, codes of practice and guidance, in different countries. This makes this a complex subject.

The annual retail sales of the nutritional supplement industry in the United States of America increased from $8.8 billion in 1994 to $18.8 billion in 2003, an increase of 115% of which a sizable proportion was spent on “sports supplements” [1,2]. The exponential increase in supplement sales can be attributed to aggressive marketing by manufacturers, rather than the development of more effective nutritional supplements [3,4]. As a result of the complex legislation governing supplements in most countries, the companies can make unsubstantiated claims about the efficacy of the supplement [5,6]. Furthermore, the accuracy of the labelling often goes unchallenged, therefore any effects of the supplement may be due to contaminants or adulterants in these products not reflected on the label [7-13]. Contamination may be defined as divergence from the information provided on the label. It may occur for various reasons, ranging from accidental to incidental [14].

The way in which the supplement industry is managed, is in stark contrast to the drug industry, which has strict legislation and control. Divergence between food and drug laws has generated “grey” areas with regard to the “voluntary” declaration of “all” content in a specific nutritional supplement product, making the product manufacture chain difficult to deal with or even subject to appropriate law enforcement [14]. Although some Consumer Protection and Anti-Doping Agencies have requested stricter report requirements for supplement manufacturers and tougher penalties for repeat offenders, legislation remains unchanged [11,15,16].

Therefore the aim of this study was to determine how consumers of nutritional supplement products acquire information to assist their purchasing decisions [17-23]. People between the age of 19–40 years, who were either moderately physically active or competitive were asked to complete a self-administered questionnaire. They were questioned about the container label information and information other than container labelling sources, which influenced their purchasing decisions for nutritional supplements. It was intended that this information would assist in providing an evidence-based solution to the problem of poorly regulated labels on nutritional supplements.

## Methods

### Materials and study population

The self-administered questionnaire was approved by the University of Cape Town, Faculty of Health Sciences Human Research Ethics Committee (HREC REF 346/2012). Written approval was obtained from the various institutions or organisation, where target and convenient samples/groups were identified and followed up as part of the recruitment process. The specific sites were the University of Cape Town (UCT), the Cape Peninsula University of Technology (CPUT), and the University of the Western Cape (UWC) sport halls, and at the holding camps for the 2012 South Africa Olympic (Pretoria) and Paralympic (Johannesburg) teams, respectively, prior to their departure to the 2012 London Olympic Games. All participants who were present at the recruitment sites and gave informed consent were eligible for the study.

Subjects who were physically challenged, visually impaired or deaf were assisted by the principal investigator or by professional support to ensure appropriate communication. The questionnaire consisted of seven, closed and open-ended questions from a defined list of statements. Question statements provided were basic and the participants had the option of *yes* or *no* answers. From statements with defined options, participants could choose the respective categories ranging from, *strongly influenced* to *no influence*. The categories of physical activity were explained to the participants. Competitively physically active was defined as organised sport at a high performance level. Moderately physically active was defined as organised sport at a social and/or recreational level.

The questionnaire covered the following specific areas, (i) general information about participants such as gender, age and the level of physical activity, (ii) had participants purchased nutritional supplement in the last 12 months, (iii) what information on the container label influenced the purchase of nutritional supplements, and (vi) what had influenced the purchase of nutritional supplements if it was not the information on the label. No importance was placed on any of the specific categories listed. The participants were asked to complete the questionnaire as accurately as possible, and to the best of their ability. The questionnaire took approximately 2–5 minutes to complete, and was administered in various similar classroom settings, over the period July to September 2012.

The data and processing of the returned questionnaires was captured using Windows-based Microsoft® Office Excel 2003 SP 1 (Excel © 1985–2003 Microsoft Corporation). Statistica Version 10 (copyright © Stat Soft, Inc. 1984–2011) was used to calculate the descriptive statistics. Data are expressed as the mean ± standard deviation (SD).

## Results

### Level of physical activity and age of participants

Seventy-seven participants described their physical activity as moderate, whilst 181 of the participants stated that they are competitively active. Only one participant indicated that he was inactive and was therefore excluded from further analysis. The mean age and standard deviation (SD) for the participants whose physical activity was classified as either moderate or competitive is presented in Table 1.

**Table 1:** Level of physical activity and age participants

**Level of physical activity**	**No. of participants**	**Participants’age (years)**
**Moderate**	77	29.5 ± 9.9
**Competitive**	181	27.6 ± 8.1
**Total group**	258	28.2 ± 8.7

*SD* standard deviation.

### Physical activity and gender comparison

Of the overall cohort (n = 258), 159 were male and 99 were female participants. Fifty males indicated they were moderately active and 109 were competitively active. For the females 27 were moderately active and 72 were competitively active, respectively. The ages of the respective male and female groups based on level of physical activity are presented in Table 2.

**Table 2:** Physical activity and gender comparison

**Level of physical activity**	**No. of participants**	**Participants’ age (years)**
**Male (total)**	159	29.5 **±** 9.4
*Moderate*	50	31.0 **±** 10.1
*Competitive*	109	28.8 **±** 9.0
**Females (total)**	99	26.1 **±** 6.9
*Moderate*	27	26.9 **±** 8.7
*Competitive*	72	25.8 **±** 6.1
**All groups (total)**	258	28.2 **±** 8.7

### Purchase of nutritional supplements

**Figure 1:**
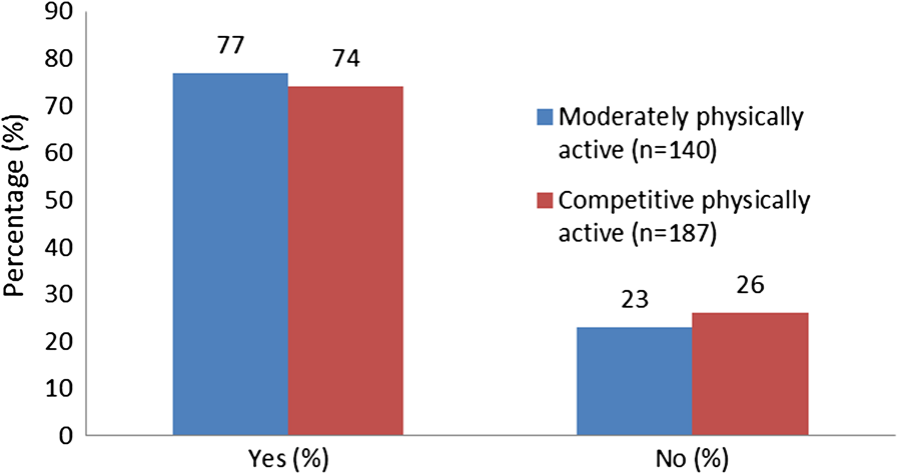
Purchase of nutritional supplements in last 12 months.

The responses of the competitively physically active group indicated that 139 (74%) had purchased nutritional supplements in the previous 12 months, whilst 48 (26%) had not purchased nutritional supplements in this period. For the moderately physical active group, 61 (77%) indicated that they had purchased nutritional supplements, whilst 79 (23%) indicated that they had not purchased nutritional supplements in the previous 12 months. These data are shown in Figure 1.

### Information on container label

When the data of the whole group (n = 195) that had purchased nutritional supplement products were analysed, 132 (68%) indicated that they were influenced by information on the container label, and 63 (32%) indicated that their purchase of a nutritional supplement product was not based on information on the product label.

The results were similar (i.e. whether the information on the label of the supplement product influenced their purchase) when the moderately and competitively physically active groups were compared (Table 3).

**Table 3:** Influence of container label in purchase

**Level of physical activity**	**No. of participants**	**Yes**	**No**
**Whole group**	195 (100%)	132 (68%)	63 (32%)
*Moderate*	58 (100%)	37 (64%)	21 (36%)
*Competitive*	135 (100%)	93 (69%)	42 (31%)

Percentage (%) of respective groups in brackets.

#### Information on the container label that influenced purchase of nutritional supplements

**Figure 2:**
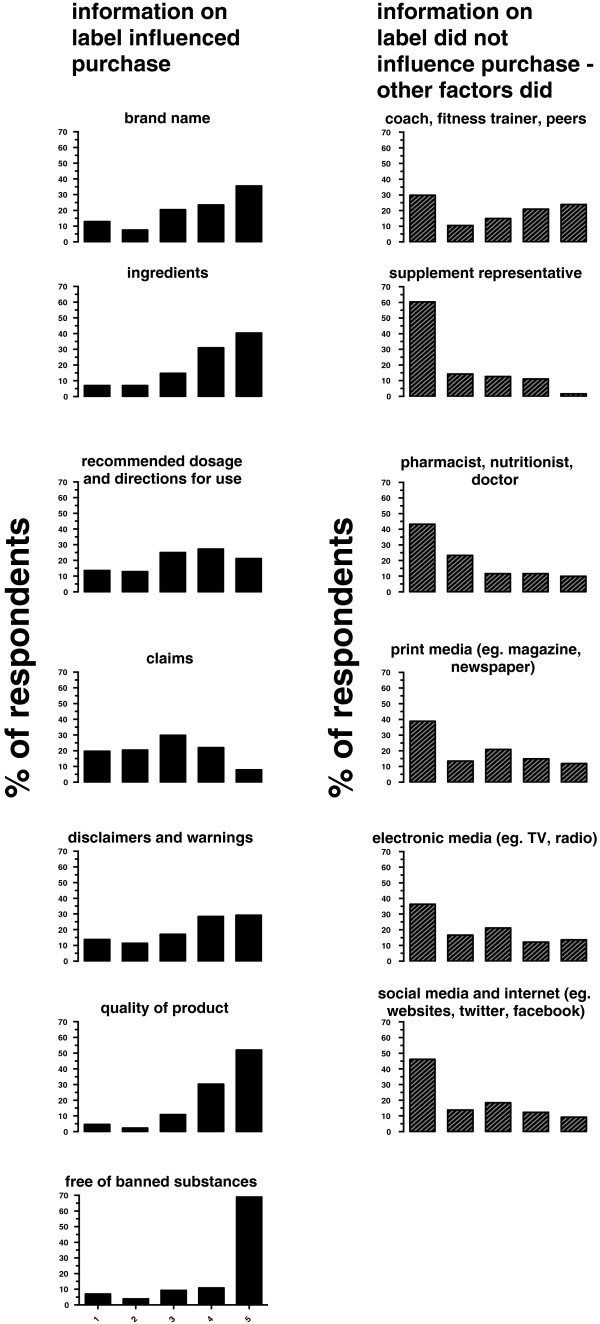
**Information influencing purchasing choice.** 1- Absolutely no influence, 2- partially no influence, 3- uncertain, 4-moderately influenced 5- strongly influenced. The bar-charts on the left-hand side illustrate the information on the container label. The bar charts on the right-hand side report on factors, other than the information on the container label.

The self-administered questionnaire provided the five defined categories, (i) *absolutely no influence*, (ii) *partially no influence*, (iii) *uncertain*, (iv) *moderately influenced* and (v) *strongly influenced* that could be selected by participants for the respective container label information. The results are presented for (n = number of respondents), (a) brand name (n = 132), (b) ingredients (n = 129), (c) recommended dosage and directions for use (n = 132), (d) claims (n = 127), (e) disclaimers and warnings (n = 123), (f) quality of product (n = 129), and (g) free of banned substances. (n = 129). Participants could respond to specific labelling information and the extent this influenced their decision making, in the defined categories. The overall results are presented in Figure 2.

The pertinent findings of information on the container label that *strongly influenced* purchase of nutritional supplements based on the percentage number of respondents were, brand name 36% (n = 47), ingredients 40% (n = 52), recommended dosage and directions for use 21% (n = 28), claims 8% (n = 10), disclaimers and warnings 29% (n = 36), quality of product 52% (n = 67), and, free of banned substance(s) 69% (n = 89).

#### Information not on container label

The results are presented in the following areas (n = number of respondents), (a) coach, gym and/or fitness trainer, and fellow athletes (n = 67), (b) supplement representatives (n = 63), (c) pharmacist, dietician, nutritionist and doctors (n = 60), (d) print media (n = 67), (e) electronic media (n = 66), and (f) social media and the internet (n = 65). The five categories were scored as previously described, ranging from *absolutely no influence* to *strongly influenced.* The pertinent findings of information not on container label that *strongly influenced* purchase of nutritional supplements based on the percentage number of respondents were; coach, gym and/or fitness trainer, and fellow athletes 24% (n = 16), supplement representatives 2% (n = 1), pharmacist, dietician, nutritionist and doctors 10% (n = 6), print media 12% (n = 8), electronic media 14% (n = 9), and, social media and the internet 9% (n = 6).

## Discussion

The main finding of this study, with particular emphasis on moderately physically active and competitive participants, was that close to 70% of the respondents who purchased supplements were *strongly influenced* by container label information, that stipulated that the nutritional supplement product is free of banned substances. The second finding is that just over 50% of the respondents attach importance to the quality of the nutritional supplement product information on the container label. The third finding, is that about 40% of the respondents were *strongly influenced* by the ingredients on the labels, when they purchased nutritional supplements. Brand name (36%), disclaimers and warnings (29%), recommended dosage and directions for use (21%), and claims (8%) accounted for the other reasons influencing the purchase.

These findings are important as they show the information that is pertinent to people who purchase nutritional supplements, and who base their purchasing decision(s) on container label information. The absence of specific information with reference to “free of banned substances” is an important determinant in the purchase of nutritional supplement products. Further, many interactions exist between drugs and nutrition. In many instances drugs and nutrients use similar sites for absorption and are metabolised and excreted through the same organs [24,25]*.* Not presenting this information on the label may have dire consequences to the health and wellness of the consumer of such products, particularly when the supplement contains a prohibited substance through contamination or incorrectly labelled product. The complexity of the issue in sport, is that in addition to potential consequences to health and drug-interaction, contaminated or incorrectly labelled products may lead to inadvertent doping. This information points to the level and degree of concern that consumers of nutritional supplements would have, if not all product content is declared on the container label.

Furthermore, concealing information about a nutritional supplement product that is adulterated or contaminated, would impact the consumer’s decision to purchase a product. These findings therefore also point to the importance of independent laboratory screen testing of all nutritional supplement products for contaminants and/or adulterants on a regular, and batch-to-batch basis.

### Implication of findings - container label information

#### Brand name

The trend based on the study cohort, shows a steady increase in the number of respondents who were not *influenced* (13%) by the brand name to those who were strongly *influenced* (36%) by brand name. This finding could be exploited by marketers to encourage consumer brand loyalty.

#### Ingredients

Seven percent of respondents were *not influenced* by ingredient content compared to 40% who were *strongly influence*d by ingredient content. The importance of ingredients in this study is supported by research in publications of Cohen [26] and Lachenmeier [25]. Their research raises concern for the evasive behaviour of some manufacturers, who make it difficult to laboratory detect undeclared ingredients, by incorporating pharmaceutical analogues into their products. That these analogues have never been studied in humans, further adds to the complexity of the situation [26]. Misleading advertising may also cause consequences for the health of the consumers due to chemical risk, if non-approved ingredients are used or contamination occurs [25,27].

#### Recommended dosage and directions for use

The trend based on the study cohort showed that there was similarity in the number of respondents who were *uncertain (25%)*, *moderately (27%),* and *strongly influenced (21%)* with respect to recommended dosage and directions for use. This observation raises concern and shows that the respondents may not necessary have clarity of understanding, of the importance and possible consequence of this type of information, if not applied correctly.

#### Claims

The findings in this study raise concern due to the greater percentage of respondents who were *uncertain* (30%), if claims information influenced their decision-making. The work of Lachenmeier [25] and Petroczi [14] support the finding of this study, stating that advertisement with claims, typically health or disease claims are misleading or scientifically unproven, despite regulations prohibiting such statements [14,25]. Further, owing to the market characteristics, enforcement is less straightforward than it is for food or feed, yet the consequences could be more severe as supplements are concentrated [14].

#### Disclaimers and warnings

The trend based on the study cohort showed similarity for the number of respondents *moderately (30%) and strongly (29%) influenced by disclaimer and warning information in making their purchasing decisions.*

#### Quality of product

The quality of product trend, showed similarity to that for brand name and ingredients information, indicating an exponential increase in the number of respondents who were *not influenced* (5%) to those who were *strongly influenced* (52%). This finding is supported by the work of Cohen [26] that shows an emerging risk to public health, due to the potential overuse of supplements and the paucity of enforcement. Petroczi [14] further reports of supplements that were found to contain more or less than the amount of substance on the label, and for the presence for contamination as a result of poor quality control measures during the product manufacture stage.

#### Free of banned substance(s)

The number of respondents who were not influenced (7%) by the supplement being free of banned substances when they were deciding to purchase, was lower than those who were *strongly influenced* (69%). The importance and assurance of a product being “free of banned substance(s)” by the study cohort studied is encouraging. What is alarming in the context of this finding, is that a recent paper showed that for the consumer to make informed choice there was a need to alert those consuming nutritional supplements of the potential for banned substances being present in products [16,28]. Of the products labels assessed in that particular study, only 5% had information on, “The presence of banned substances in the supplements”. A supporting paper that assessed the regulations, legislation, and claims associated with nutritional supplements products, concluded that consumer protection provisions should promote greater levels of policy development, regulatory enforcement, and consumer education [6,15,29]. Petroczi [14] also reports on the discovery that many supplements contain hazardous substances such as illegal anabolic steroids that have serious known side effects. This is a concern, and points to the importance of having an awareness of the health risk associated with certain hormonal substances and stimulants. This awareness may lead people to opt for “natural” supplements supposedly free of such ingredients [14].

The findings other than the container label information raise awareness of the factors that *strongly influences* the purchasing of nutritional supplement products. An important finding was that coaches, gym and/or fitness trainers, and fellow athletes (24%), have a *greater influence* on the choice of nutritional supplement products, than that of a Pharmacist, Dietician, Nutritionist and Doctors (10%). This is contrary to the recommendations of Meltzer [30], which provided a practical guide to the use of nutritional supplements in South Africa, specifically stating that fitness coaches and conditioning staff should not prescribe supplements.

### Implication of findings - other than the container label information

#### Coach, gym and/or fitness trainer, and fellow athletes

This study showed a steady increase in the number of respondents who were partially *not influenced* (10%) by a coach, gym, fitness trainer and/or fellow athletes to those who were strongly *influenced* (24%). This finding is supported by the work of Petroczi [14] that raises public health concerns for supplement consumers who frequently consume beyond the knowledge or remit of clinical practitioners, who act unilaterally or upon the advice of non-experts, such as fellow athletes or coaches. This scenario of poorly informed decision-making, is likely to be more prevalent in the general population where numerous supplements are often taken at levels considerably above the recommended daily allowance [14].

#### Supplement representatives

Information provided by supplement representative showed an exponential decrease in the number of respondents who were *not influenced* (60%) to those who were strongly *influenced* (2%). This is encouraging as it shows that supplement representatives did not have much influence in the purchase decisions of supplements in the cohort studied.

#### Pharmacist, dietician, nutritionist and doctors

There was a decreasing trend in the number of respondents who were *not influenced* (43%) by information provided by Pharmacist, Dietician, Nutritionist and Doctors to those who were strongly *influenced* (10%). Work by Cohen, which supports this finding, shows that the regulatory environment for supplements is often poorly understood by both consumers and physicians, and also because premarket testing of supplement is not a requirement by most regulators globally [31]. Further, consumers believed that supplements are approved by government agency, and also thought that governments require that labels on supplements include warnings about their potential side effects and dangers. The authors also suggest that medical doctors should maintain a high index of suspicion for using supplements, even when the components on the label are not known to cause the observed effects [26].

#### Print media

There was a steady decrease in the number of respondents who were *not influenced* (39%) by print media information (e.g. magazines, newspapers) to those who were *strongly influenced* (12%). This form of information could provide the opportunity for consumer education and awareness that will ensure informed choice, through improved knowledge.

#### Electronic media

The trend for electronic media was similar to that of print media. There was a steady decrease in the number of respondents who were *not influenced* (36%) by electronic media information (e.g. television, radio) to those who were *strongly influenced* (14%). Consumer education and awareness could also be provided via this medium to improve knowledge and so ensure informed choice.

#### Social media and the internet

The trend for social media and the internet was similar to the trend for print media and electronic media. There was a steady decrease in the number of respondents who were *not influenced* (46%) by Social media and the internet information (e.g. website, twitter, Facebook) to those who were *strongly influenced* (9%).

Internet shops are currently not controlled in depth if at all, with respect to inspections, including sampling and analysis. Yet, large proportion of the supplement market is sold exclusively via this route by companies outside the European Union (EU), and reaches consumers via the postal service. Nutritional supplements also appear to be the most problematic group of products on the internet and hence the importance for professional athletes to be careful, due to contamination or as intentional addition of substances on the World Anti-Doping Agency list [14,25,27]. Further, the majority of “sports food” marketed on the web was either found to be ineffective and misleadingly labelled, or if effective, they contained pharmaceutically active substances which are not approved for food use [25]. What these observations imply is that these are potential areas of growth to provide peer-reviewed evidenced-based information.

#### Limitations of study

With hindsight it would have been useful to have included a specific category, “parental influence in purchases”. Furthermore, the findings of this study only relate to the specific groups of moderately physically active and competitive participants between the ages of 19–40 years. It is accepted that younger and older participants, may have responded differently to the questionnaire. There may also have been different influences within the broad professional categories that were used in the questionnaire. Future research in this area should also explore gender or sport-level differences as this could further provide informative insight, on purchasing decision-making.

### Recommendations

#### Regulation and enforcement

To enhance the overall quality of supplements there needs to be a concerted effort across sovereign states and continental unions, to align regulatory frameworks and methods of surveillance.

#### Anti-doping laboratory for supplements and punitive measures

Accredited laboratories with the same status as anti-doping agency laboratories need to be introduced with adequate global standard. In all these cases, a physical sample would be required to judge whether the supplement has complied with the required safety requirements.

#### Early warning communication systems

Rapid communication system needs to be instituted where contaminated supplements are reported via monitoring and enforcement, within and across sovereign states. The communication respective platforms, should also form the basis for synergy and harmonization, that will contribute to “global” monitoring and enforcement of nutritional supplements. Further, governments should have the responsibility to equip appropriately their law enforcement capabilities, that will alert timeously of “problem products” and with the necessary rigor, have these products recalled.

#### Consumer education and awareness

National Sport and Athlete Coaching forums, Sports confederations and Olympic confederations need to provide knowledge to coaches pertaining to the potential for “supplement doping” to occur. Whilst in the current World anti-doping agency (WADA) regulations, athletes are responsible for what they consume, the consequence for “testing” positive, has negative consequence also for those associated with the “doper”, particular at the high performance level. For the general public, the brief and mandate for Consumer Forums need to be expanded. In the absence of sovereign laws and regulations in the specific area for nutritional supplements, guideline documents, position and policy statements would serve an effective implementation strategy to counter the possibility for “supplement doping”.

#### Controlled clinical trials

In the absence of controlled clinical trials, adverse health effects from dietary/nutritional supplements should be assessed from patients and doctors’ reports, and case studies. Supplements need to be scientifically tested, so that side effects are not inaccurately attributed and diagnosed, and thus leading to incorrect policies and/or warnings.

## Conclusion

The evidence in the study to assess the impact of container labelling and other sources of information on consumer purchasing decisions, highlights matters that require attention in the interest of both the nutritional supplement sector and the consumer. The evidence points to; (a) short-comings in current labelling information practices, (b) the need for practical intervention, policy development, and/or regulation, (c) opportunities to improve label and non-label information and communication, (d) how nutritional products may be marketed in the future, that is contrary to the current approach, (e) the need for the consumer to make informed choices with complete knowledge and understanding of all product content, linked to container label content, and, (f) the requirement for quality assurance laboratory “screen testing” of declared and undeclared contaminants and/or adulterants, that could have negative consequences to the consumer.

## Competing interests

The authors declare that they have no competing interests.

## Authors’ contributions

GG contributed to the design, recruitment, data collection, data analyses, interpretation and presentation, the drafting and the main writing to the paper. ML contributed to the design, data analyses, interpretation and presentation and editing of paper. All authors have read and approved the final manuscript.
